# High-Precision Machining Method of Weak-Stiffness Mirror Based on Fast Tool Servo Error Compensation Strategy

**DOI:** 10.3390/mi12060607

**Published:** 2021-05-24

**Authors:** Zelong Li, Yifan Dai, Chaoliang Guan, Jiahao Yong, Zizhou Sun, Chunyang Du

**Affiliations:** 1 College of Intelligence Science and Technology, National University of Defense Technology, 109 Deya Road, Changsha 410073, China; d20153105@163.com (Z.L.); dyf@nudt.edu.cn (Y.D.); 18302996932@163.com (Z.S.); 15573125954@sina.cn (C.D.); 2 Hunan Key Laboratory of Ultra-Precision Machining Technology, Changsha 410073, China; 3 Laboratory of Science and Technology on Integrated Logistics Support, National University of Defense Technology, 109 Deya Road, Changsha 410073, China; 4 61175 Troops, The Chinese People’s Liberation Army, Naijing 210049, China; jiahaoyong1996@163.com

**Keywords:** weak-stiffness mirror, fast tool servo, clamping error, cutting error, error compensation

## Abstract

Weak-stiffness mirrors are widely used in various fields such as aerospace and optoelectronic information. However, it is difficult to achieve micron-level precision machining because weak-stiffness mirrors are hard to clamp and are prone to deformation. The machining errors of these mirrors are randomly distributed and non-rotationally symmetric, which is difficult to overcome by common machining methods. Based on the fast tool servo system, this paper proposes a high-precision machining method for weak-stiffness mirrors. Firstly, the clamping error and cutting error compensation strategy is obtained by analyzing the changing process of the mirror surface morphology. Then, by combining real-time monitoring and theoretical simulation, the elastic deformation of the weak-stiffness mirror is accurately extracted to achieve the compensation of the clamping error, and the compensation of the cutting error is achieved by iterative machining. Finally, a weak-stiffness mirror with a thickness of 2.5 mm was machined twice, and the experimental process produced a clamping error with a peak to valley (PV) value of 5.2 µm and a cutting error with a PV value of 1.6 µm. The final machined surface after compensation had a PV value of 0.7 µm. The experimental results showed that the compensation strategy proposed in this paper overcomes the clamping error of the weak-stiffness mirror and significantly reduces cutting errors during the machining process, achieving the high precision machining of a weak-stiffness mirror.

## 1. Introduction

In the fields of aerospace equipment, optical imaging systems are usually built with weak-stiffness mirrors in order to meet the need for light weight. A specially shaped aluminum mirror is used in the infrared horizon system to realize the imaging function of the optical system [1]. The shape accuracy of the mirror surface determines the performance of the instrument, and the higher the shape accuracy, the higher the imaging resolution of the optical system. In order to meet the requirements of visible light imaging, the peak to valley (PV) value of the surface shape accuracy should be better than two λ (λ = 632.8 nm) [2]. Compared with an ordinary optical mirror, weak-stiffness mirrors are difficult to clamp and have low stiffness, which makes them very prone to clamping deformation and cutting deformation [3]. General weak-stiffness mirrors will produce a clamping deformation greater than 5 µm after clamping, which obviously cannot meet the demand of visible light imaging [4]. How to effectively solve the issues of clamping deformation and cutting deformation is the key to achieving the high-precision machining of such mirrors.

One of the main machining difficulties of weak-stiffness mirrors is that after disassembling the mirror, the mirror surface will spring back and destroy the original machined surface. The magnitude and distribution of the springback is uncertain because it is difficult to accurately control the magnitude of the clamping force. The common solution to this problem is the optimization of the clamping method for controlling the clamping deformation within an acceptable range [5]. Pan et al. analyzed the effect of clamping force on the thin-walled part deformation at different clamping positions and obtained the nonlinear relationship between contact force, contact deformation, and contact area, which is beneficial for reducing clamping deformation [6]. Miao et al. chose a low rigidity shell part as a research target, the machining process was designed and analyzed, different clamping schemes were designed for this part, and the comparison and analysis were finished by using the finite element method [7]. However, the clamping method is different for various mirrors, and it is difficult to control the clamping error within two λ. Second, the machining process of weak-stiffness mirrors will produce large cutting errors due to cutting deformation, machine motion errors, servo system tracking errors, and other factors [8,9]. Many models have been established to describe the cutting errors during the cutting process [10,11]. Liu et al. established a model of machining errors in three-axis ultra-precision lathes based on the theory of multi-body systems [12]. Zhou et al. established an orthogonal cutting force analysis model based on unequal parallel-edge shear bands and proposed a cutting force prediction algorithm [13]. However, various errors are coupled with each other in the cutting process, and it is difficult to establish an accurate model to describe these cutting errors.

Both clamping error and cutting error are randomly distributed non-rotational symmetric errors. Ordinary two-axis turning cannot realize the compensation of non-rotational symmetry errors, and slow tool servo cutting can realize the compensation of non-rotational symmetry errors, but its working frequency is low, generally only 10–20 Hz [14]. To realize the compensation of clamping errors and cutting errors, a higher working frequency is required. A fast tool servo (FTS) system is a high-frequency, high-precision machining method that is widely used in ultra-precision machining processes [15,16]. Compared to the slow tool servo, the FTS working frequency is greater than 100 Hz and has nanometer-level resolution, which can realize the cutting of micron-level complex error morphology [17]. FTS systems have been utilized for machine tool error compensation and the cutting of complex morphology. Kim et al. used an FTS system with a servo bandwidth of 100 Hz for the real-time compensation of machine tool axial errors, and completed the machining of a large aspheric off-axis aluminum mirror with a diameter of 620 mm [18]. Yu et al. used an FTS system to compensate for contour errors and to improve the machining accuracy of microarrays [19]. Few studies have been conducted on the use of fast servo tools for the cutting process of weak-stiffness mirrors, and this paper investigates the feasibility of using an FTS system to compensate for the clamping and cutting errors of weak-stiffness mirrors.

In order to improve the machining accuracy of weak-stiffness mirrors, based on the fast tool servo system, this paper proposes a compensation strategy for clamping errors and cutting errors during the turning of a weak-stiffness mirror. The structure of this paper is as follows: the second section introduces the compensation strategies for clamping errors and cutting errors. The third section introduces the simulation and analysis methods, including the FTS machining system, clamping deformation simulation, and the Z-axis working frequency analysis. The fourth section presents the experimental results and discussion. Finally, the conclusion is given in the fifth section.

## 2. Error Compensation Strategy

**Figure 1:**
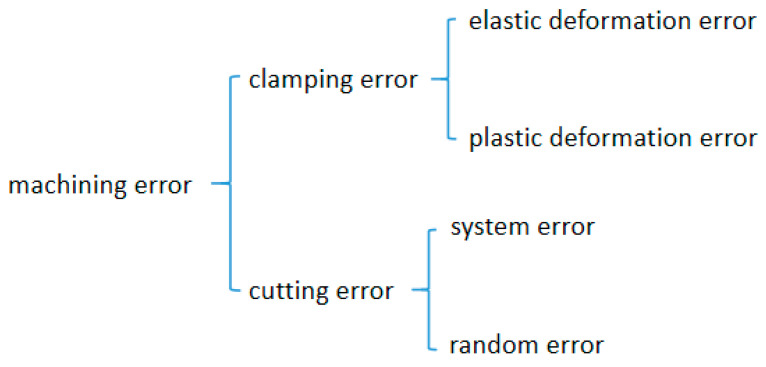
Schematic diagram of machining error distribution.

As shown in Figure 1, the machining errors of a weak-stiffness mirror are divided into clamping errors [20] and cutting errors [21]. Clamping errors includes elastic deformation errors and plastic deformation errors, while cutting errors can be divided into system errors and random errors.

### 2.1. Clamping Error Compensation Strategy

**Figure 2:**
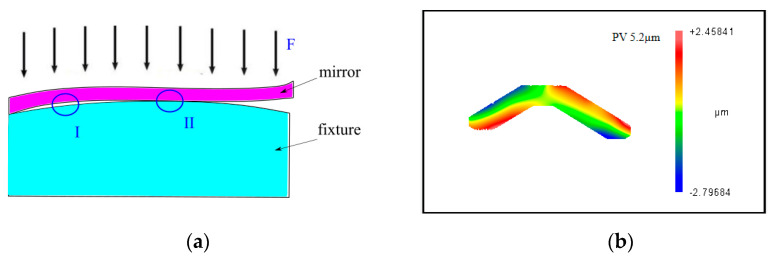
(**a**) Contact surface of the mirror and the fixture (I is the non-contact area, II is the contact area) and (**b**) the clamping deformation of a weak-stiffness mirror.

**Figure 3:**
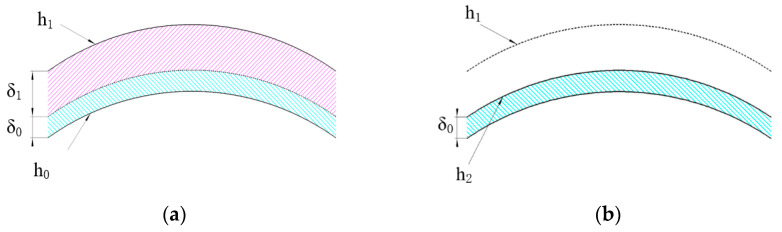
Changes in the mirror surface after clamping and removing. (**a**) The mirror surface after clamping and (**b**) the mirror surface after removing.

As the contact surface between the mirror and the fixture is not an ideal plane, there is a contact area and a non-contact area, as shown in Figure 2a. Therefore, a large clamping force is generated during the clamping process, which will lead to clamping deformation on the machined surface [22]. Figure 2b shows the clamping deformation of a weak-stiffness mirror obtained through experimental measurement. Different colors represent different heights. The maximum height was about 2.4 µm, the minimum height was about −2.8 µm, and the PV value was about 5.2 µm. Clamping deformation includes elastic deformation and plastic deformation. Elastic deformation will recover after the removal of the fixture, while plastic deformation is a permanent deformation that cannot be recovered. As shown in Figure 3a, h_0_ is the mirror surface before clamping. After clamping, the elastic deformation δ_1_ and the plastic deformation δ_2_ are produced. h_1_ is the mirror surface after clamping. As in Figure 3b, after removing the fixture, the elastic deformation δ_1_ recovers, while the plastic deformation δ_2_ is still present. h_2_ is the mirror surface after springback.

Based on the deformation process in Figure 3, this paper proposes a clamping deformation compensation strategy. Table 1 lists the relevant symbols. The compensation strategy is mainly for accurately obtaining the elastic deformation of the mirror surface, and then correcting the desired machining trajectory z_0_ according to the elastic deformation. First, in order to accurately obtain the elastic deformation, the clamping method is optimized to a certain extent, and then the clamping deformation is controlled in the elastic region by finite element simulation. Therefore, there is no plastic deformation δ_2_. The specific process of the simulation is described in Section 3.2. Then, the elastic deformation δ_1_ can be obtained by measuring the mirror surface before and after clamping, as in Equation (1):

δ_1_ = h_1_ − h_0_ (1)

Second, the obtained elastic deformation δ_1_ is used to correct the desired machining trajectory z_0_, and then the first machining trajectory z_1_ is obtained as Equation (2). The mirror is always clamped during the whole machining process, which means that there is always an elastic deformation δ_1_ on the mirror surface. After the fixture is removed, the elastic deformation of the mirror surface will spring back, which will offset the corrected elastic deformation δ_1_, and the equivalent machining trajectory z_3_ after springback is consistent with the ideal machining trajectory z_0_, so as to achieve clamping error compensation. The final equivalent machining trajectory z_3_ after springback is given by Equation (3):

z_1_ = z_0_ + δ_1_ (2)

z_3_ = z_1_ − δ_1_ = z_0_ (3)

### 2.2. The Cutting Error Compensation Strategy

**Figure 4:**
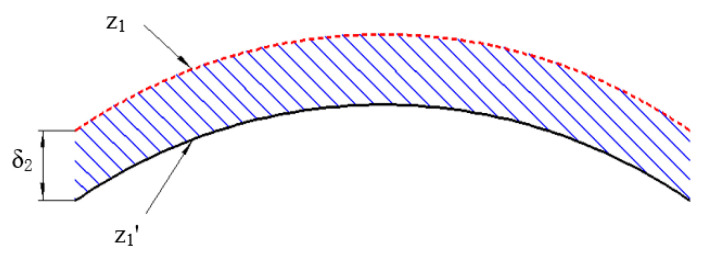
Cutting error.

Section 2.1 introduces the compensation for the clamping error, but a weak-stiffness mirror will also produce a larger cutting error, mainly due to cutting deformation in the actual machining process. As shown in Figure 4, according to the description in Section 2.1, the machining trajectory for the first machining is z_1_, but the actual machining trajectory is z_1_’ under the influence of the cutting error δ_2_.

There are many factors that can cause cutting errors. Some studies have shown that cutting errors can be divided into repeatable system errors and random errors caused by random factors such as machine tool status changes or tool wear under similar operating conditions [23]. Additionally, when the machine tool is in good condition and the operation is accurate, cutting errors are mainly repetitive system errors.

**Figure 5:**
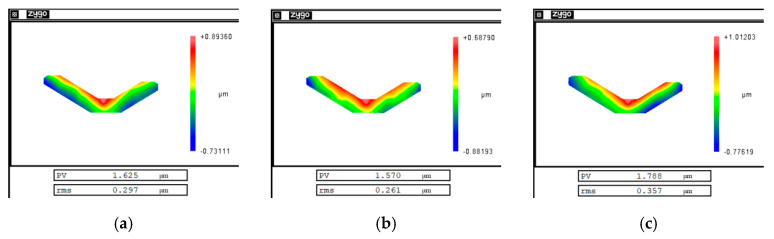
Morphological distribution of cutting errors at (**a**) the first cutting, (**b**) the second cutting, and (**c**) the third cutting.

In order to verify that the cutting error was mainly a repetitive system error, repeated cutting experiments were performed on the same mirror under the same experimental conditions and machining trajectory. The experiment was carried out with an ordinary ultra-precision machine tool, and the machining result was measured with a vertical interferometer. The experiment speed was 300 r/min and the cutting depth was 1.4 µm. The cutting error produced by each cutting experiment was obtained by subtracting the machining trajectory from the actual measurement results. Figure 5 shows the distribution of cutting errors produced by each cutting experiment. Table 2 summarizes the PV and root mean square (RMS) values of the cutting error in each experiment. It can be seen from the experimental results that the magnitude and distribution of the cutting error generated by each cutting experiment were almost the same. The cutting errors were repeatable.

Based on the above analysis, the compensation strategy for cutting errors is proposed. Firstly, the first machining is performed according to the description in Section 2.1, and the first machining trajectory is z_1._ Then, the mirror is kept in the clamped state and the actual machining trajectory z_1_’ is obtained by measuring the machining results. The cutting error δ_2_ generated during this machining process is obtained by Equation (4). Then, the obtained cutting error is used to correct the first machining trajectory and obtain the second machining trajectory z_2_, as shown in Equation (5). Ignoring the influence of random error, it is believed that the second machining will produce a cutting error of the same magnitude and distribution as the first machining. The actual second machining trajectory z_2_’ will be equal to the first machining trajectory z_1_, thereby realizing the compensation of the cutting error, as shown in Equation (6).

δ_2_ = z_1_ − z_1′_ (4)

z_2_ = z_1_ + δ_2_ (5)

z_2′_ = z_2_ − δ_2_ = z_1_ (6)

In summary, the compensation of clamping errors and cutting errors is achieved by two machining operations, respectively. The first machining compensates for the clamping errors and the second machining compensates for the cutting errors produced in the first machining. After considering the cutting error, the final equivalent trajectory z_3_ is still equal to the ideal machining trajectory z_0_.

z_3_ = z_2′_ − δ_1_ = z_1_ − δ_1_ = z_0_ (7)

## 3. Simulation and Analysis

### 3.1. FTS Machining System

Different from the ordinary machine tool error compensation, the clamping errors and cutting errors in this paper are randomly distributed errors on the order of microns. To realize this error compensation, the machining Z-axis needs to have C, X, and Z-axis linkage capability and a high working frequency.

**Figure 6:**
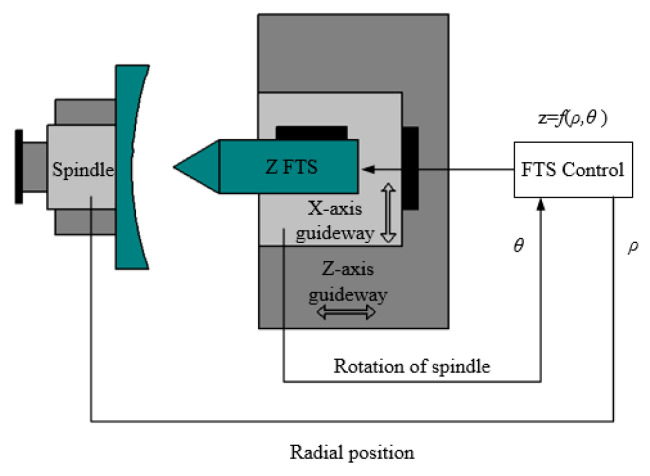
FTS machining system.

In order to meet the basic requirements for error compensation, an ultra-precision cutting system based on FTS technology is established, as shown in Figure 6. The ultra-precision cutting system consists of the Z-axis, X-axis, and C-axis of the ultra-precision machine and FTS. During processing, the workpiece rotates with the C-axis, and the X-axis feeds along the radial direction of the workpiece. The FTS calculates the feed amount z required for the current machining position according to the current spindle angle *θ* and the X-axis position (i.e., the radial position of the workpiece) *ρ*, and then drives the diamond tool to perform a quantitative feed motion along the Z-axis to cut the workpiece. Since the feed amount z of the fast tool servo system is a function of the spindle angle and the position of the X-axis z = *f*(*ρ*,*θ*), it can realize machining with arbitrarily distributed errors. The working frequency of the Z-axis of the system is greater than 500 Hz, and the working stroke is greater than 100 µm.

**Figure 7:**
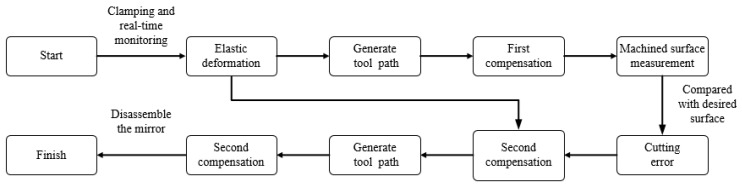
Flow chart of the error compensation experiment.

The basic process of the error compensation experiment is shown in Figure 7. It mainly includes the following steps: (a) real-time monitoring of the clamping process to extract the elastic deformation. First, measure the mirror surface before clamping. The mirror surface will change after clamping. After stabilization, measure the mirror surface after clamping. (b) Generate the first machining trajectory based on the extracted elastic deformation. (c) Compare the result after the first compensation machining with the desired machining result to obtain the cutting error. (d) Superimpose the cutting error on the basis of the first compensation machining and generate the second machining trajectory. (e) Disassemble the mirror and obtain the final result.

### 3.2. Clamping Deformation Simulation

According to the description in Section 2.1, the clamping deformation error needs to be controlled in the elastic region to accurately extract the elastic deformation error. In this section, we ensure that the clamping deformation is only elastic deformation through finite element simulation when the magnitude and distribution of the clamping deformation are known.

**Figure 8:**
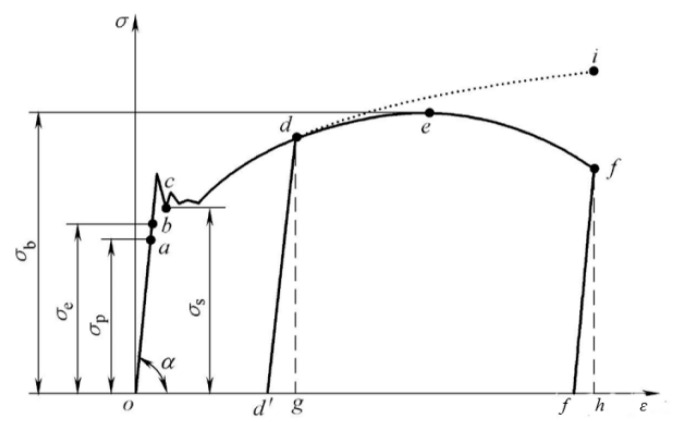
Stress–strain curve.

As shown in Figure 8, when the clamping force acts on the surface of the aluminum alloy, with the yield limit σ_s_ as the boundary, the work done by the clamping gradually accumulates in the material as elastic deformation energy and then produces elastic deformation. With the gradual increase in the degree of deformation, the accumulation of plastic deformation energy starts to occur after passing the yield point b, and then plastic deformation occurs [24]. According to the Mises yield criterion, Mises considers yield as an invariant state of the material, independent of the stress coordinate system. When the equivalent stress reaches the yield limit σ_s_, the material begins to produce plastic deformation. The equivalent stress can be obtained by Equation (8):

(8) $$\bar{\sigma }=\frac{1}{\sqrt{2}}\sqrt{{\left(\sigma_{1}-\sigma_{2}\right)}^{2}+{\left(\sigma_{2}-\sigma_{3}\right)}^{2}+{\left(\sigma_{3}-\sigma_{1}\right)}^{2}}$$

**Figure 9:**
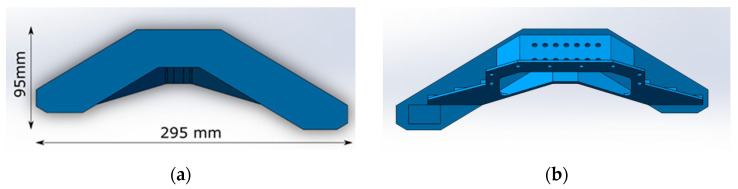
Infrared horizon mirror 3D model: (**a**) mirror machining surface and (**b**) mirror clamping surface.

**Figure 10:**
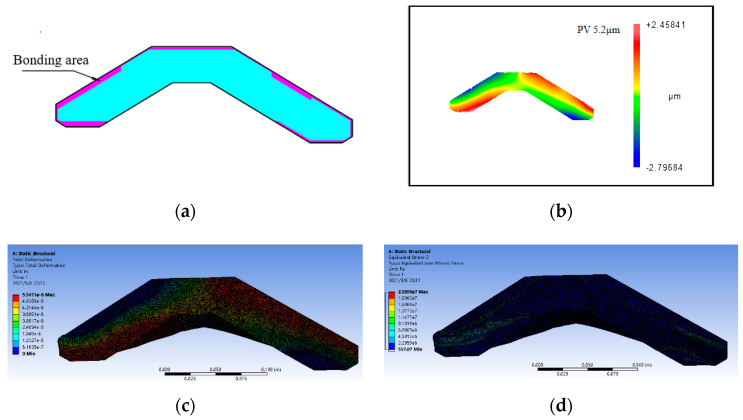
Clamping deformation simulation of a weak-stiffness mirror: (**a**) bonding area; (**b**) measurement results of clamping deformation; (**c**) machining surface displacement distribution map; and (**d**) machining surface stress distribution map.

The experimental object is an infrared horizon mirror, as shown in Figure 9. The mirror is 295 mm long, 95 mm wide, 2.5 mm thick, and made of aluminum alloy with a yield limit σ_s_ 275 Mpa. In order to reduce the clamping force, the mirror is clamped by bonding, using low-stress extreme ultraviolet glue (uv-50). The glue will harden only after being irradiated by ultraviolet light. The bonding area is shown in Figure 10a. Figure 10b shows the magnitude and distribution of the clamping deformation obtained by measuring the mirror surface before and after clamping. Due to the complexity of the stresses generated by bonding, forced displacements are applied to the machined mirror surface based on the measured clamping deformation. Then, the stress distribution on the machined surface is obtained by the Mises yield criterion, as shown in Equation (8) [25]. The finite element simulation software was ansys workbench 19.0, the element was solid186, and the number of elements was 642485. Figure 10c is a displacement distribution diagram of the machining surface and Figure 10d is a stress distribution diagram of the machining surface.

From the experimental and simulation results, it can be seen that the clamping force of the extreme ultraviolet glue produced a clamping deformation with PV value of 5 µm on the machined surface. As shown in Figure 10d, it can be seen that the maximum stress was 20 MPa, which is far less than the yield limit σ_s_ of 275 MPa of the aluminum alloy material. According to the analysis in Figure 8, we can reach the conclusion that the mirror surface only has elastic deformation.

### 3.3. The Analysis of Z-Axis Working Frequency

#### 3.3.1. Spatial Curve Extension Algorithm

**Figure 11:**
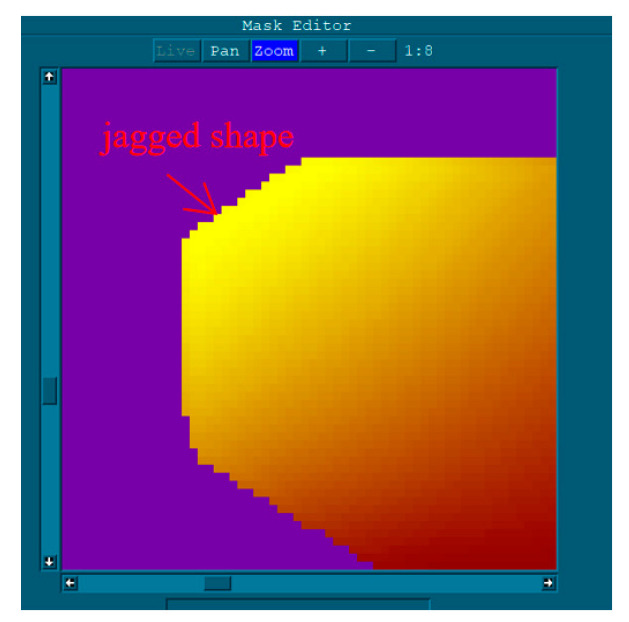
24 inch interferometer measurement results (resolution 0.00066 m).

To make the Z-axis work more smoothly and to reduce the Z-axis working frequency, the spatial curve extension algorithm is used to extend the Z-axis machining trajectory before machining. Since the infrared horizon mirror is not a regular circle, there must be a part of the machining trajectory outside the mirror, and the tool position outside the mirror needs to be processed. In addition, the mirror has beveled edges and is large. Due to the limited resolution of the interferometer measurement, the edge will have a jagged shape, as shown in Figure 11.

**Figure 12:**
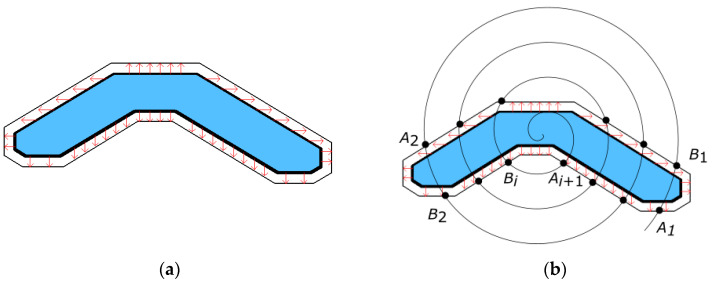
Spatial curve extension algorithm: (**a**) edge data extension and (**b**) data extension outside the boundary.

The space curve extension algorithm is divided into two steps. First, the edge data are extended by 3 pixel points (about 2 mm) along the vertical and horizontal directions, as shown in Figure 12a. Then, the data are extended along the spiral trajectory based on the cubic spline interpolation algorithm. Figure 12b shows the projection of the machining trajectory on the plane of the cylindrical coordinate system. The boundary point entering the mirror is *A(Aρ,Aθ,Az),* and the boundary point leaving the mirror is *B*(*Bρ,Bθ,Bz*). The tool moves from the point *B_i_*(*Bρ_i_,Bθ_i_,Bz_i_*) to the point *A_i_*_+1_(*Aρ_i+_*_1_,*Aθ_i_*_+1_*,Az_i+1_*). P*_i_^j^*(*Pρ_i_^j^,Pθ_i_^j^,Pz_i_^j^*) is a certain interpolation point of the curve *B_i_A_i+1_*, which is calculated by Equation (9):

(9) $$\begin{array}{l} Pz_{i}^{j}=\frac{(L+2l_{i}^{j}){\left(L-l_{i}^{j}\right)}^{2}}{L^{3}}Bz_{i}+\frac{(3L-2l_{i}^{j}){\left(l_{i}^{j}\right)}^{2}}{L^{3}}Az_{i+1}\\ +\frac{(l_{i}^{j}){\left(L-l_{i}^{j}\right)}^{2}}{L^{2}}Bk_{i}+\frac{{\left(l_{i}^{j}\right)}^{2}(l_{i}^{j}-L)}{L^{2}}Ak_{i+1} \end{array}$$

where *L* is the arc length of the projection of the space curve *B_i_A_i_*_+1_ on the plane of the column coordinate system, *l_i_^j^* is the arc length of the projection of the curve *B_i_P_i_^j^* on the plane of the column coordinate system, *Bk_i_* is the slope of the boundary point *B_i_*, and *Ak_i_*_+1_ is the slope of the boundary point *A*_i+1_. Since the highest item does not exceed 3 times in Equation (9), it is guaranteed that the Z value of the interpolated curve segment will not rise and fall more than 2 times. 

#### 3.3.2. The Analysis of Z-Axis Working Frequency

**Figure 13:**
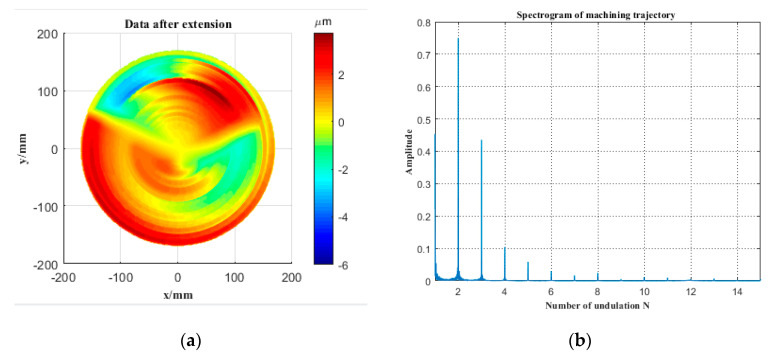
(**a**) Data after extension and (**b**) a spectrogram of the machining trajectory.

Figure 13a is the result of the mirror surface data after extension. Figure 13b shows the spectrum of the machining trajectory after extension, which is obtained by spectrum analysis. N is the number of undulations in a rotation cycle of the Z-axis. According to Equation (10), when the spindle speed n = 300 r/min and the number of fluctuations N = 15 in a rotation period, the Z-axis working frequency *f* = 75 Hz. The working frequency of the FTS system built in this paper is greater than 500 Hz, which meets the needs of use.

(10) $$f=\frac{N\times n}{60}$$

where *f* is the Z-axis working frequency Hz, n is the spindle speed r/min, and N is the number of undulations of the Z-axis in one rotation cycle.

## 4. Experimental Results and Discussion

### 4.1. Experimental Results

**Figure 14:**
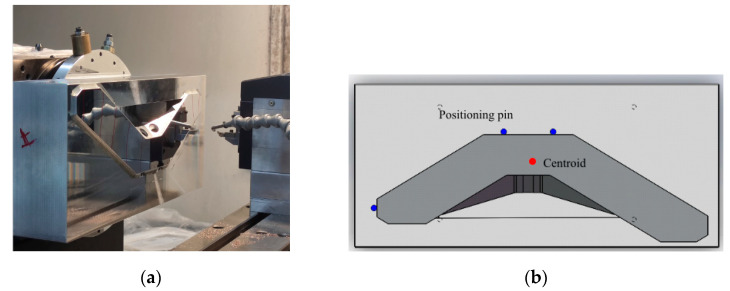
(**a**) FTS system and (**b**) a schematic diagram of the mirror and fixture.

Figure 14a shows the FTS machining system used in this experiment, with a maximum working stroke of 0.5 mm and a working frequency of 500 Hz. Figure 14b shows the experimental workpiece and fixture, positioned by the positioning pin holes on both sides. The center of mass of the workpiece coincides with the center of rotation of the spindle. A diamond tool with a radius of 0.2 mm is used. The spindle speed is 300 r/min, the finishing X-axis feed rate is 1.2 mm/min, and the depth of cut is 1.4 µm.

**Figure 15:**
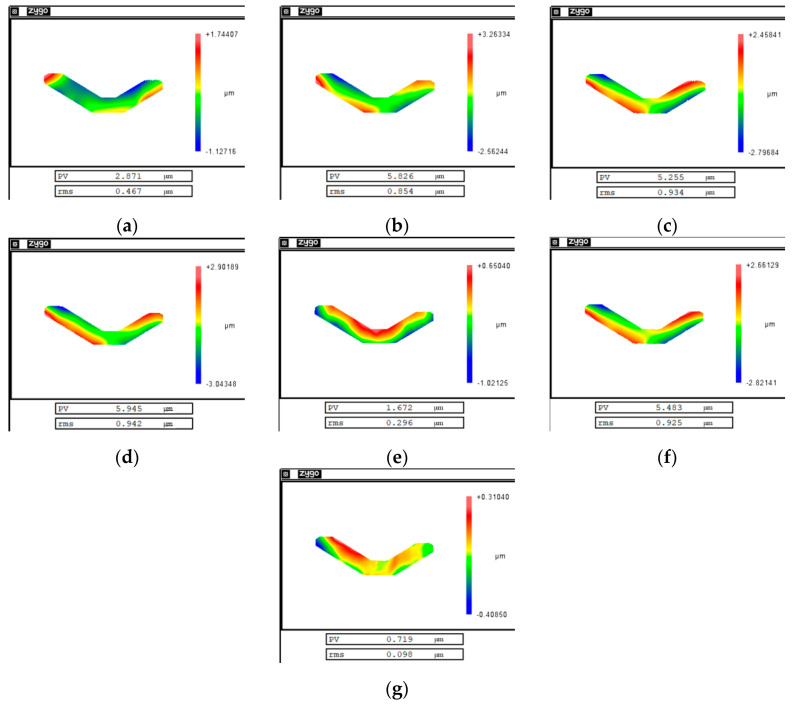
Experimental results: (**a**) is the measurement result before clamping; (**b**) is the measurement result after clamping; (**c**) is the elastic deformation error; (**d**) is the measurement result after the first machining; (**e**) is the cutting error; (**f**) is the measurement result after the second machining; and (**g**) is the final measurement result after disassembling the mirror.

**Figure 16:**
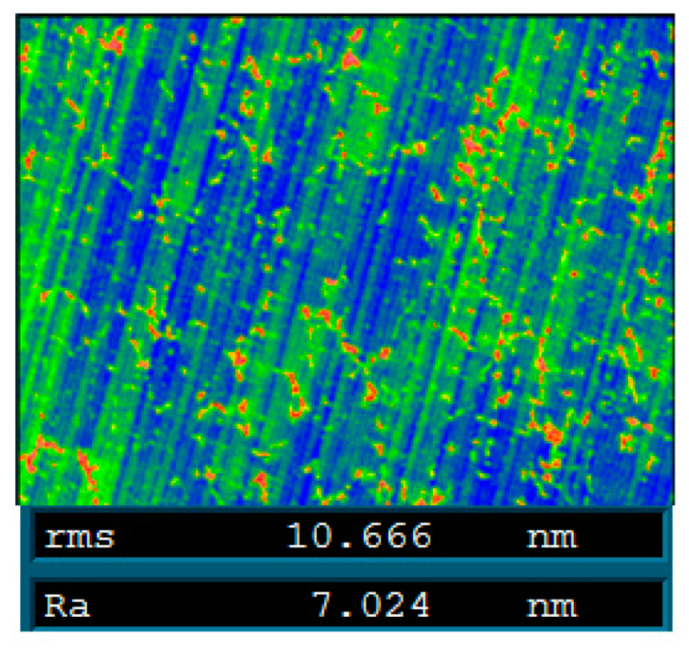
Surface roughness of the mirror.

Figure 15 shows the measurement results of the mirror surface during the experiment. As shown in Figure 15c,e, there was a large clamping error and cutting error generated in this experiment. The PV value of the clamping error was 5.2 µm and the PV value of the cutting error was 1.6 µm. The PV value of the final mirror surface was 0.7 µm after two compensations, as shown in Figure 15g. Additionally, the final surface roughness Ra was better than 10 nm, as shown in Figure 16.

### 4.2. Discussion

The experimental results show that the machining method based on the FTS error compensation strategy proposed in this paper can effectively overcome the clamping errors and cutting errors of a weak-stiffness mirror and significantly improve the machining accuracy. This machining method has strong applicability and can solve the machining problems of most mirrors with low stiffness and easy deformation. Based on the experimental results and the compensation strategy, there are a few points to discuss.

**Figure 17:**
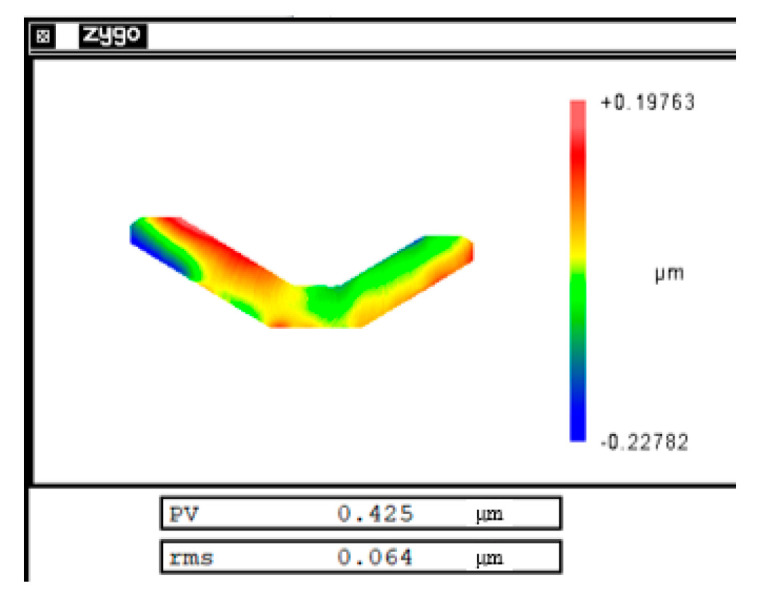
Cutting error after compensation.

(1) In order to extract the elastic deformation of the mirror accurately, the compensation strategy proposed in this paper needs to control the clamping deformation in the elastic region. Through real-time monitoring and theoretical simulation, it ensures that the clamping deformation generated by each clamping is elastic. This method can be applied to most weak-stiffness mirrors. However, it may not be possible to control the clamping deformation in the elastic region for some mirrors that are very difficult to clamp or are very prone to plastic deformation. In this case, some adjustments can be made to the compensation strategy. First, the relationship between the elastic deformation δ_1_ and the plastic deformation δ_0_ needs to be obtained, as in Equation (11), and K is the scale factor. Then, based on Equation (11), the elastic deformation of the workpiece can still be obtained based on the measurement of the surface before clamping h_0_ and the surface after clamping h_1_, as in Equation (12) and Equation (13). The subsequent compensation strategy remains the same. To accurately obtain the scale factor K, we need to further investigate the elastic–plastic deformation theory.
  δ_0_ = Kδ_1_ (11)
  h_1_ − h_0_ = δ_0_ + δ_1_ = (1 + K)δ_1_ (12)
  δ_1_ = (h_1_ − h)/K (13)
(2) In this paper, cutting errors are compensated by iterative machining. It was verified by repeatability experiments that the cutting errors were dominated by system errors under the same experimental conditions. Figure 17 shows the cutting error after compensation. The PV value of the cutting error was reduced from 1.6 µm to 0.4 µm. In order to maintain a good surface roughness on the machined surface, each machining time was about 2.5 h. During the second compensated machining, the machine condition was changed due to the long machining time and the change of the ambient temperature, which led to a large random error. It can be assumed that the residual error of the mirror surface was mainly the random error generated by the two machining processes. By reducing the machining time and controlling the ambient temperature to maintain the machine in a similar state during the two machining processes, the machining accuracy of the weak-stiffness mirror can be further improved.
(3) This paper gives the calculation of the Z-axis working frequency at different spindle speeds, which is instructive. Different weak-stiffness mirrors produce different magnitudes and distributions of errors and require different Z-axis working frequencies. The researcher can select a suitable FTS system based on the calculation result of Equation (10).

## 5. Conclusions

This paper proposes a high-precision machining method for weak-stiffness mirrors based on the FTS error compensation strategy. The elastic deformation of the mirror is accurately extracted by real-time monitoring and clamping deformation simulation. The compensation of the clamping error is realized by correcting the machining trajectory. Additionally, the cutting error is significantly reduced by iterative machining. The Z-axis working frequency required for realizing the error compensation was quantitatively calculated by a spatial curve extension algorithm and spectrum analysis. Based on the FTS system, a weak-stiffness mirror was machined twice. The final PV value of the machined surface was 0.7 µm. It effectively solved the clamping error with a PV value of 5.2 µm and a cutting error with a PV value of 1.6µm generated during the machining of the weak-stiffness mirror. The experimental results show that the machining method proposed in this paper effectively solves the clamping errors of weak-stiffness mirrors and significantly reduces the cutting errors during the machining process. The machining method proposed in this paper is instructive and can be widely applied to the machining of weak-stiffness mirrors.

## Figures and Tables

**Table 1:** Some nomenclature.

Symbol	Meaning
z_0_	the desired machining trajectory
z_1_	the first machining trajectory
z_1′_	the actual first machining trajectory
z_2_	the second machining trajectory
z_2′_	the actual second machining trajectory
z_3_	the final equivalent machining trajectory
h_0_	the mirror surface before clamping
h_1_	the mirror surface after clamping

**Table 2:** Cutting error repeatability test results.

Experimental Result	First Experiment	Second Experiment	Third Experiment
PV value	1.625 µm	1.570 µm	1.788 µm
RMS value	0.297 µm	0.261 µm	0.357 µm
